# Vibration-assisted upconversion of molecular luminescence induced by scanning tunneling microscopy

**DOI:** 10.1186/1556-276X-8-204

**Published:** 2013-05-01

**Authors:** Kuniyuki Miwa, Mamoru Sakaue, Hideaki Kasai

**Affiliations:** 1 Department of Applied Physics, Osaka University, 2-1 Yamadaoka, Suita, Osaka 565-0871, Japan

**Keywords:** Upconversion, Molecular luminescence, Scanning tunneling microscope, Surface plasmon, Molecular vibration, Exciton-plasmon coupling, Nonequilibrium Green’s function method

## Abstract

We investigate the effects of coupling between a molecular exciton, which consists of an electron and a hole in a molecule, and a surface plasmon (exciton-plasmon coupling) on the electron transitions of the molecule using nonequilibrium Green’s function method. Due to the exciton-plasmon coupling, excitation channels of the molecule arise in the energy range lower than the electronic excitation energy of the molecule. It is found that the electron transitions via these excitation channels give rise to the molecular luminescence and the vibrational excitations at the bias voltage lower than the electronic excitation energy of the molecule. Our results also indicate that the vibrational excitations assist the emission of photons, whose energy exceeds the product of the elementary charge and the bias voltage, (upconverted luminescence).

## Background

Light emission from molecules on metal substrates induced by tunneling current of a scanning tunneling microscope (STM) has attracted much attention owing to its fascinating new physics and its wide applicability in molecular nano-electronics and nano-optics [1-6]. Since surface plasmons localized near the tip-substrate gap region generate an intense electromagnetic field, effects of the interaction between the intense electromagnetic field and the transition moments of the molecular excitations and de-excitations are expected to occur [7-11]. Therefore, in STM-induced light emission (STM-LE) from the molecule on the metal substrate, the interplay between the excitation/de-excitation processes of the molecule and the surface plasmons plays an important role. To understand this from a microscopic point of view, there is a need to investigate the dynamics of the molecule and the surface plasmons within the framework of quantum many-body theory. We have recently investigated the effects of coupling between a molecular exciton, which consists of an electron and a hole in the molecule, and the surface plasmon (exciton-plasmon coupling) on the luminescence properties of the molecule and the surface plasmons with the aid of the nonequilibrium Green’s function method [12]. Our results have shown that the luminescence spectral profiles of the molecule and the surface plasmons can be strongly influenced by the interplay between their dynamics resulting from the exciton-plasmon coupling.

**Figure 1:**
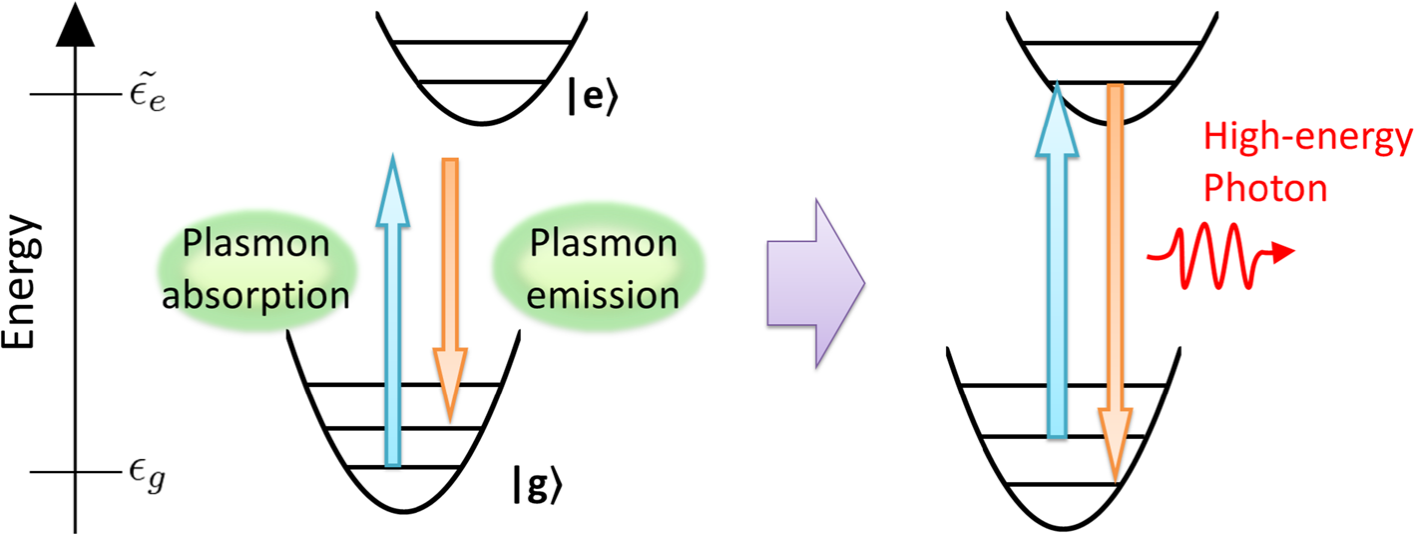
**Schematic diagram of mechanism for occurrence of the upconverted luminescence.** Horizontal lines in each parabola denote vibrational sublevels where |*g*〉 and |*e*〉 denote the electronic ground and excited states, respectively. The electronic excitation and de-excitation of the molecule are induced by the absorption and emission of the surface plasmon, respectively. These electron transitions are accompanied by the vibrational excitations, and the vibrational excitations assist the occurrence of the upconverted luminescence.

Recently, the emission of photons, whose energy exceeds the product of the elementary charge and the bias voltage *eV*_bias_, (upconverted luminescence) has been observed. Generally, when the excitations of the samples are induced by one tunneling electron, the energy of emitted photons is considered to be less than *eV*_bias_. This condition is called the quantum cutoff condition and has been satisfied in most experiments [5,9,10]. However, in recent studies of STM-LE from tetraphenylporphyrin (TPP) molecules on metal substrates, the upconverted luminescence has been observed despite the fact that *eV*_bias_ is lower than the highest occupied molecular orbital-lowest unoccupied molecular orbital (HOMO-LUMO) gap energy *E*_*ex*_[13]. One of the possible mechanisms is as follows: the electronic excitation (de-excitation) of the molecule is induced by the absorption (emission) of the surface plasmon; these electron transitions are accompanied by the excitations of the molecular vibration [14], and the vibrational excitations assist the occurrence of the upconverted luminescence (Figure 1). However, the detailed mechanism for the occurrence of these electron transitions at *eV*_bias_ < *E*_*ex*_ has not yet been clarified. In this study, we investigate the effects of the exciton-plasmon coupling on the electron transitions of the molecule at *eV*_bias_ < *E*_*ex*_ using nonequilibrium Green’s function method. We found that the electron transitions of the molecule occur via the excitation channels resulting from the exciton-plasmon coupling. The results also show that the vibrational excitations assist the occurrence of the upconverted luminescence.

## Methods

We consider a model which includes the electronic ground (excited) state of the molecule |*g*〉 (|*e*〉). The electron on the molecule interacts with the molecular vibrations and the surface plasmons. The Hamiltonian of the system is

(1) $$\begin{array}{lll} \;H= & \underset{m=g,e}{\sum }\epsilon_{m}c_{m}^{†}c_{m}+\hbar \omega_{0}b^{†}b+\hbar \omega_{p}a^{†}a\; & \;\\ & +\underset{\beta }{\sum }\hbar \omega_{\beta }b_{\beta }^{†}b_{\beta }+MQ_{b}c_{e}^{†}c_{e}\; & \;\\ & +V\left(ac_{e}^{†}c_{g}+\mathrm{H.c.}\right)+\underset{\beta }{\sum }U_{\beta }Q_{b}Q_{\beta },\; \end{array}$$

where $c_{m}^{†}$ and *c*_*m*_(*m* = *e*, *g*) are creation and annihilation operators for an electron with energy *ϵ*_*m*_ in state |*m*〉. Operators *b*^†^ and *b* are boson creation and annihilation operators for a molecular vibrational mode with energy $\hbar \omega_{0}$; *a*^†^ and *a* are for a surface plasmon mode with energy $\hbar \omega_{p}$, and $b_{\beta }^{†}$ and *b*_*β*_ are for a phonon mode in the thermal phonon bath, with *Q*_*b*_ = *b* + *b*^†^ and $Q_{\beta }=b_{\beta }+b_{\beta }^{†}$. The energy parameters *M*, *V*, and *U*_*β*_ correspond to the coupling between electronic and vibrational degrees of freedom on the molecule (electron-vibration coupling), the exciton-plasmon coupling, and the coupling between the molecular vibrational mode and a phonon mode in the thermal phonon bath.

By applying the canonical (Lang-Firsov) transformation [15], *H* becomes

(2) $$\begin{array}{lll} \;\tilde{H}= & \epsilon_{g}c_{g}^{†}c_{g}+\tilde{\epsilon_{e}}c_{e}^{†}c_{e}+\hbar \omega_{0}b^{†}b+\hbar \omega_{p}a^{†}a\; & \;\\ & +\underset{\beta }{\sum }\hbar \omega_{\beta }b_{\beta }^{†}b_{\beta }\; & \;\\ & +V\left(aX^{†}c_{e}^{†}c_{g}+\mathrm{H.c.}\right)+\underset{\beta }{\sum }U_{\beta }Q_{b}Q_{\beta },\; \end{array}$$

where *X* = exp[-*λ*(*b*^†^ - *b*)], $\tilde{\epsilon_{e}}=\epsilon_{e}-M^{2}/(\hbar \omega_{0})$ and $\lambda =M/(\hbar \omega_{0})$.

The luminescence spectra of the molecule are expressed in terms of Green’s function of the molecular exciton on the Keldysh contour [16], which is defined as

(3) $$\begin{array}{lll} & \;L(\tau ,\tau^{\prime })\; & \;\\ & \;=\frac{1}{i\hbar }{\langle T_{C}\{c_{g}^{†}(\tau )c_{e}(\tau )c_{e}^{†}(\tau^{\prime })c_{g}(\tau^{\prime })\}\rangle }_{H}\; & \;\\ & \;=\frac{1}{i\hbar }{\langle T_{C}\{c_{g}^{†}(\tau )c_{e}(\tau )X(\tau )c_{e}^{†}(\tau^{\prime })c_{g}(\tau^{\prime })X^{†}(\tau^{\prime })\}\rangle }_{\tilde{H}},\; \end{array}$$

where 〈⋯ 〉_*H*_ and ${\langle \cdots \;\rangle }_{\tilde{H}}$ denote statistical average in representations by system evolution for *H* and $\tilde{H}$, respectively. *τ* is the Keldysh contour time variable, and *T*_*C*_ is the time ordering along the Keldysh contour.

By assuming the condition of stationary current, the distribution function *N*_pl_ of the surface plasmons excited by inelastic tunneling between the tip and the substrate is given by

(4) $$N_{\text{pl}}(\omega )=\left\{\begin{array}{ll} T_{\text{pl}}\left(1-\left|\frac{\hbar \omega }{eV_{\text{bias}}}\right|\right), & \left|\hbar \omega \right|<\left|eV_{\text{bias}}\right|\\ 0, & \text{others} \end{array}\right),$$

where *T*_pl_ is a coefficient related to the current amplitude due to the inelastic tunneling [17]. We calculate *L* according to the calculation scheme previously reported by us [12]. The spectral function and the luminescence spectra of the molecule are defined by the relations,

(5) $$\begin{array}{ll} A_{L}(\omega ) & =-\frac{1}{\Pi }I\left[L^{r}(\omega )\right],\; \end{array}$$

(6) $$\begin{array}{ll} B_{L}(\omega ) & =-\frac{1}{\Pi }I\left[L^{<}(\omega )\right],\; \end{array}$$

where *L*^*r*^ and *L*^<^ are the retarded and lesser projection of *L*.

The parameters are given so that they correspond to the experiment on the STM-LE from TPP molecules on the gold surface [13]: $\left(\tilde{\epsilon_{e}}-\epsilon_{g}\right)=1.89\;\text{eV}$, $\hbar \omega_{0}=0.16\;\text{eV}$, and $\hbar \omega_{p}=2.05\;\text{eV}$. The statistical average is taken for temperature *T* = 80 K [13]. The square of *λ* is reported to be 0.61 on the basis of first-principles calculations [18]. The parameter *U*_*β*_ is given so that the molecular vibrational lifetime due to the coupling to the thermal phonon bath is 13 ps [13]. A Markovian decay is assumed for the surface plasmon so that the plasmon lifetime for *V*=0 eV becomes 4.7 fs [13,18]. The coefficient *T*_pl_ is set in the range of 10^-4^ to 10^-2^, where the tunneling current is *I*_*t*_ = 200 pA, and an excitation probability of the surface plasmons per electron tunneling event is considered to be in the range of 10^-2^ to 1.

## Results and discussion

**Figure 2:**
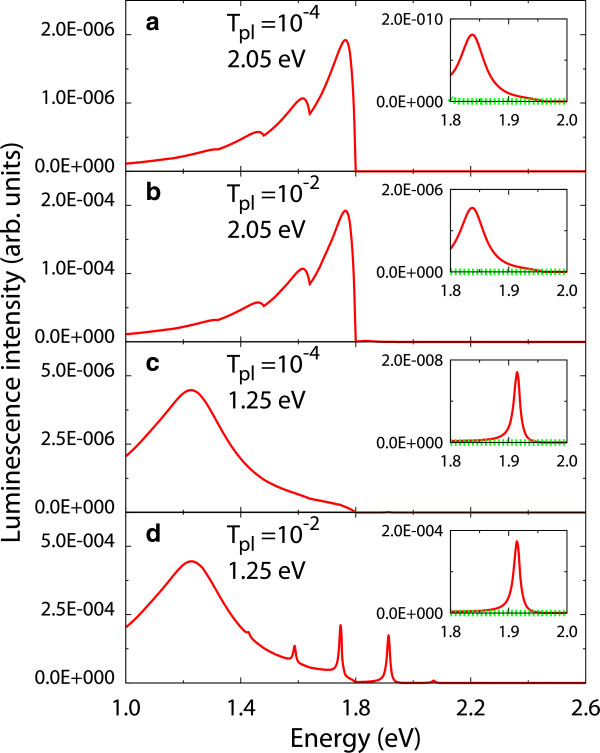
**Luminescence spectra of the molecule *B***_***L ***_**at the bias voltage *V***_**bias **_**= 1.8 V.** Insets: red solid and green dotted lines show luminescence spectra for vibrational state in nonequilibrium and thermal equilibrium, respectively. Here, (**a**) *T*_pl_ = 10^-4^ and $\hbar \omega_{p}=2.05\;\text{eV}$, (**b**) *T*_pl_ = 10^-2^ and $\hbar \omega_{p}=2.05\;\text{eV}$, (**c**) *T*_pl_ = 10^-4^ and $\hbar \omega_{p}=1.25\;\text{eV}$, and (**d**) *T*_pl_ = 10^-2^ and $\hbar \omega_{p}=1.25\;\text{eV}$. The exciton-plasmon coupling is *V* = 0.10 eV.

Figure 2 shows the luminescence spectra of the molecule *B*_*L*_ at the bias voltage *V*_bias_ = 1.8 V. Although the product of the elementary charge and the bias voltage *eV*_bias_ is lower than the HOMO-LUMO gap energy $\left(\tilde{\epsilon_{e}}-\epsilon_{g}\right)$, the molecular luminescence is found. The results indicate that the electron transitions of the molecule occur at this bias voltage. A peak structure with a long tail is observed in the energy range higher than *eV*_bias_ = 1.8 eV. The contribution of the vibrational excitations can be found in comparison with the vibrational state in thermal equilibrium, where the molecular vibration with the energy $\hbar \omega_{0}=0.16\;\text{eV}$ is distributed according to the Bose distribution function at *T* = 80 K, and therefore, the molecular vibration is almost in the ground state.

The dependence of luminescence spectra on *T*_pl_ and $\hbar \omega_{p}$ is also shown in Figure 2. The luminescence intensity increases as *T*_pl_ increases. The luminescence intensity in the energy range lower than *eV*_bias_ is proportional to *T*_pl_, and the intensity of the upconverted luminescence is proportional to the square of *T*_pl_. As the energy of the surface plasmon mode $\hbar \omega_{p}$ is shifted to the low-energy side, the luminescence intensity increases. This increase is attributed to the fact that since the energy of the surface plasmon mode is lower than *eV*_bias_, the electron transitions in the molecule in the energy range lower than *eV*_bias_ are enhanced by the surface plasmons.

**Figure 3:**
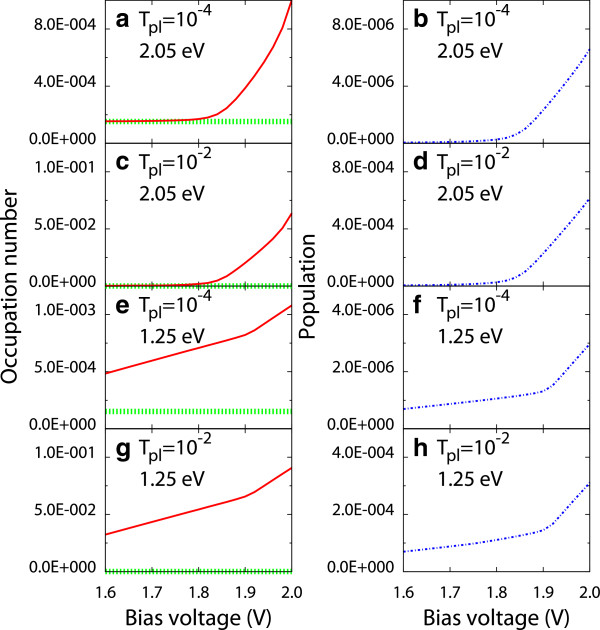
**Bias voltage dependence of the vibrational occupation number and the population of the molecular exciton.** Red solid and green dashed lines refer to the vibrational occupation number for vibrational state in nonequilibrium and thermal equilibrium, respectively. The blue dashed-dotted line refers to the population of the molecular exciton. Here, (**a**, **b**) *T*_pl_ = 10^-4^ and $\hbar \omega_{p}=2.05\;\text{eV}$, (**c**, **d**) *T*_pl_ = 10^-2^ and $\hbar \omega_{p}=2.05\;\text{eV}$, (**e**, **f**) *T*_pl_ = 10^-4^ and $\hbar \omega_{p}=1.25\;\text{eV}$, and (**g**, **h**) *T*_pl_ = 10^-2^ and $\hbar \omega_{p}=1.25\;\text{eV}$. The exciton-plasmon coupling is *V* = 0.10 eV.

Figure 3 shows the bias voltage dependence of the vibrational occupation number $n_{b}={\langle b^{†}b\rangle }_{\tilde{H}}$ and the population of the molecular exciton $n_{e}={\langle c_{e}^{†}c_{g}X^{†}c_{g}^{†}c_{e}X\rangle }_{\tilde{H}}$. It is confirmed that the vibrational excitations occur at *V*_bias_ = 1.8 V. Thus, the vibrational excitations assist the occurrence of the upconverted luminescence. The slope of *n*_*e*_ changes at *V*_bias_ of approximately 1.85 eV for $\hbar \omega_{p}=2.05\;\text{eV}$ (Figure 3b,d) and at *V*_bias_ of approximately 1.90 eV for $\hbar \omega_{p}=1.25\;\text{eV}$ (Figure 3f,h). At this bias voltage, the excitation channels of the molecule increase. The slope of the difference between *n*_*b*_ and the vibrational occupational number for the vibrational state in thermal equilibrium *n*_*b*,TE_ changes at the same bias voltage. Thus, the vibrational excitations are accompanying the electron transitions of the molecule.

**Figure 4:**
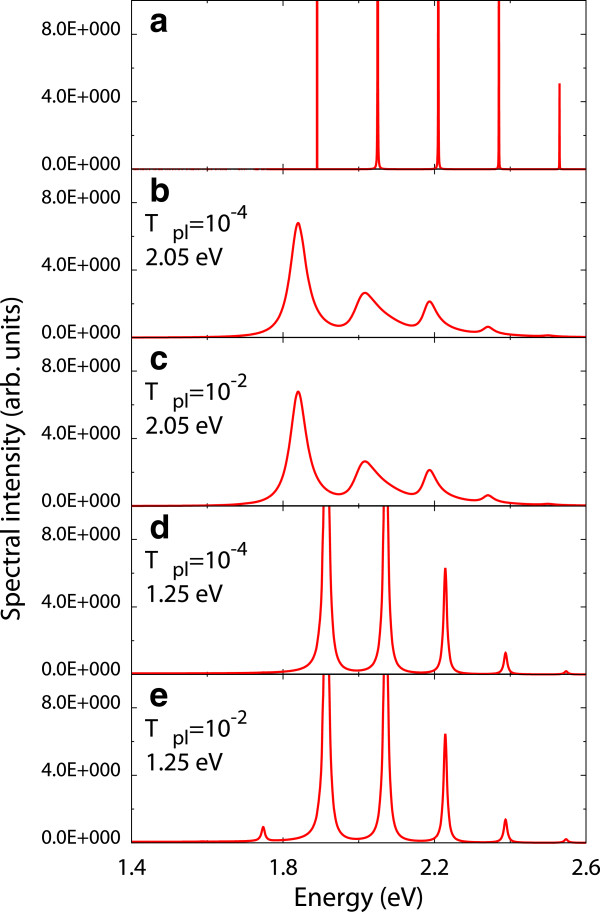
**Spectral functions of the molecule for (a) *V *= 0.0 eV and (b to e) *V *= 0.1 eV.** The bias voltage is *V*_bias_ = 1.8 V. Here, (**b**) *T*_pl_ = 10^-4^ and $\hbar \omega_{p}=2.05\;\text{eV}$, (**c**) *T*_pl_ = 10^-2^ and $\hbar \omega_{p}=2.05\;\text{eV}$, (**d**) *T*_pl_ = 10^-4^ and $\hbar \omega_{p}=1.25\;\text{eV}$, and (**e**) *T*_pl_ = 10^-2^ and $\hbar \omega_{p}=1.25\;\text{eV}$.

To analyze the mechanism for the occurrence of the electron transitions accompanied by the vibrational excitations at $V_{\text{bias}}<\left(\tilde{\epsilon_{e}}-\epsilon_{g}\right)/e$, the spectral function of the molecule *A*_*L*_ is shown in Figure 4. Due to the exciton-plasmon coupling *V*, the position and the width of the peaks in *A*_*L*_ are shifted and broadened, respectively. The spectral intensities are found in the energy range lower than $\left(\tilde{\epsilon_{e}}-\epsilon_{g}\right)$. It indicates that the excitation channels of the molecule arise in this energy range. Thus, the electron transitions of the molecule occur via the excitation channels resulting from the exciton-plasmon coupling and give rise to the vibrational excitations.

## Conclusion

The exciton-plasmon coupling has a strong influence on the luminescence property of the molecule. The excitation channels of the molecule arise even in the energy range lower than the HOMO-LUMO gap energy $\left(\tilde{\epsilon_{e}}-\epsilon_{g}\right)$. It is found that the electron transitions of the molecule via these excitation channels give rise to the molecular luminescence and the vibrational excitations at the bias voltage $V_{\text{bias}}<\left(\tilde{\epsilon_{e}}-\epsilon_{g}\right)/e$. Our results also indicate that the vibrational excitations assist the occurrence of the upconverted luminescence.

## Abbreviations

HOMO-LUMO: Highest occupied molecular orbital-lowest unoccupied molecular orbital; STM: Scanning tunneling microscope; STM-LE: Scanning tunneling microscope-induced light emission.

## Competing interests

The authors declare that they have no competing interests.

## Authors’ contributions

KM and MS conceived the idea, designed the study, analyzed the data, and drafted the manuscript. HK supervised and gave suggestions on the study. All authors read and approved the final manuscript.
